# Case Report: Epstein-Barr virus-positive follicular lymphoma with high PD-L1 expression

**DOI:** 10.3389/fimmu.2025.1696940

**Published:** 2025-10-09

**Authors:** Xue Zheng, Xuhao Wang, Yu Pan, Xiaolong Sui, Guohua Yu

**Affiliations:** ^1^ Department of Pathology, Yantai Yuhuangding Hospital, Yantai, Shandong, China; ^2^ Department of Pathology, Haiyang Hospital of Traditional Chinese Medicine, Yantai, Shandong, China

**Keywords:** follicular lymphoma, high PD-L1 expression, mechanisms, treatment, Epstein-Barr virus-positive

## Abstract

**Background:**

Follicular lymphoma is a common B-cell lymphoma and is classified as an indolent lymphoma, more frequently seen in middle-aged and elderly individuals. In recent years, considerable research has been conducted on follicular lymphoma; however, cases of Epstein-Barr virus (EBV)-positive follicular lymphoma remain rare. Notably, follicular lymphoma with EBV positivity accompanied by high PD-L1 expression has not been reported.

**Case presentation:**

We present a case of EBV-positive follicular lymphoma with high PD-L1 expression and analyze its clinicopathological features. The patient was treated with R-CHOP chemotherapy combined with the anti-CD20 antibody (rituximab), achieving favorable therapeutic outcomes. This case provides valuable insights for the pathological diagnosis and treatment of EBV-positive follicular lymphoma, as well as the role of EBV infection in PD-L1 expression. Furthermore, it raises the question of whether high PD-L1 expression suggests that immunotherapy could serve as another potential treatment strategy for EBV-positive follicular lymphoma.

## Introduction

Follicular lymphoma (FL) is a prevalent subtype of indolent lymphoma in Western countries and ranks as the second most common form of non-Hodgkin’s lymphoma, following diffuse large B-cell lymphoma. Clinically, FL often presents with generalized lymphadenopathy, bone marrow involvement, and splenomegaly, while extranodal involvement is uncommon (1).

Histologically, FL is defined by a follicular growth pattern consisting of centrocytes and centroblasts. Alongside purely follicular architecture, a mixed pattern of follicular and diffuse growth is also observed, whereas a completely diffuse growth pattern is rare (2).Tumorigenesis typically includes an asymptomatic preclinical phase, during which premalignant B-lymphocytes harboring the t (14,18) chromosomal translocation accumulate additional genetic alterations within the germinal centers, resulting in clonal evolution (3).

The International Agency for Research on Cancer (IARC) has classified Epstein-Barr virus (EBV), Hepatitis C virus (HCV), Human Immunodeficiency Virus Type 1 (HIV-1), Kaposi’s Sarcoma-associated Herpesvirus (KSHV), Human T-cell Lymphotropic Virus Type 1 (HTLV-1), and Helicobacter pylori as carcinogenic to humans, with sufficient evidence linking them to specific lymphoma types. However, only HCV has shown a definitive association with FL (4). Reports exploring the correlation between EBV infection and FL are limited.

Although FL is typically incurable, standard first-line therapies are associated with high response rates and prolonged remission in the majority of patients (5). Nevertheless, FL frequently recurs, and early relapse or transformation into a more aggressive lymphoma is associated with poorer prognosis. We report a case of EBV-positive grade 3B follicular lymphoma, in which the tumor cells exhibited high PD-L1 expression. The patient responded well to chemotherapy with the R-CHOP chemotherapy combined with anti-CD20 antibody (Rituximab), which may to some extent suggest a potential association between EBV infection and FL, as well as the association between EBV infection and high PD-L1 expression. It further confirms that the standard initial treatment for FL is also applicable to EBV-associated FL. Additionally, it is plausible that the presence of high PD-L1 expression may offer a potential supplementary treatment strategy for refractory follicular lymphoma.

## Case description

**Figure 1:**
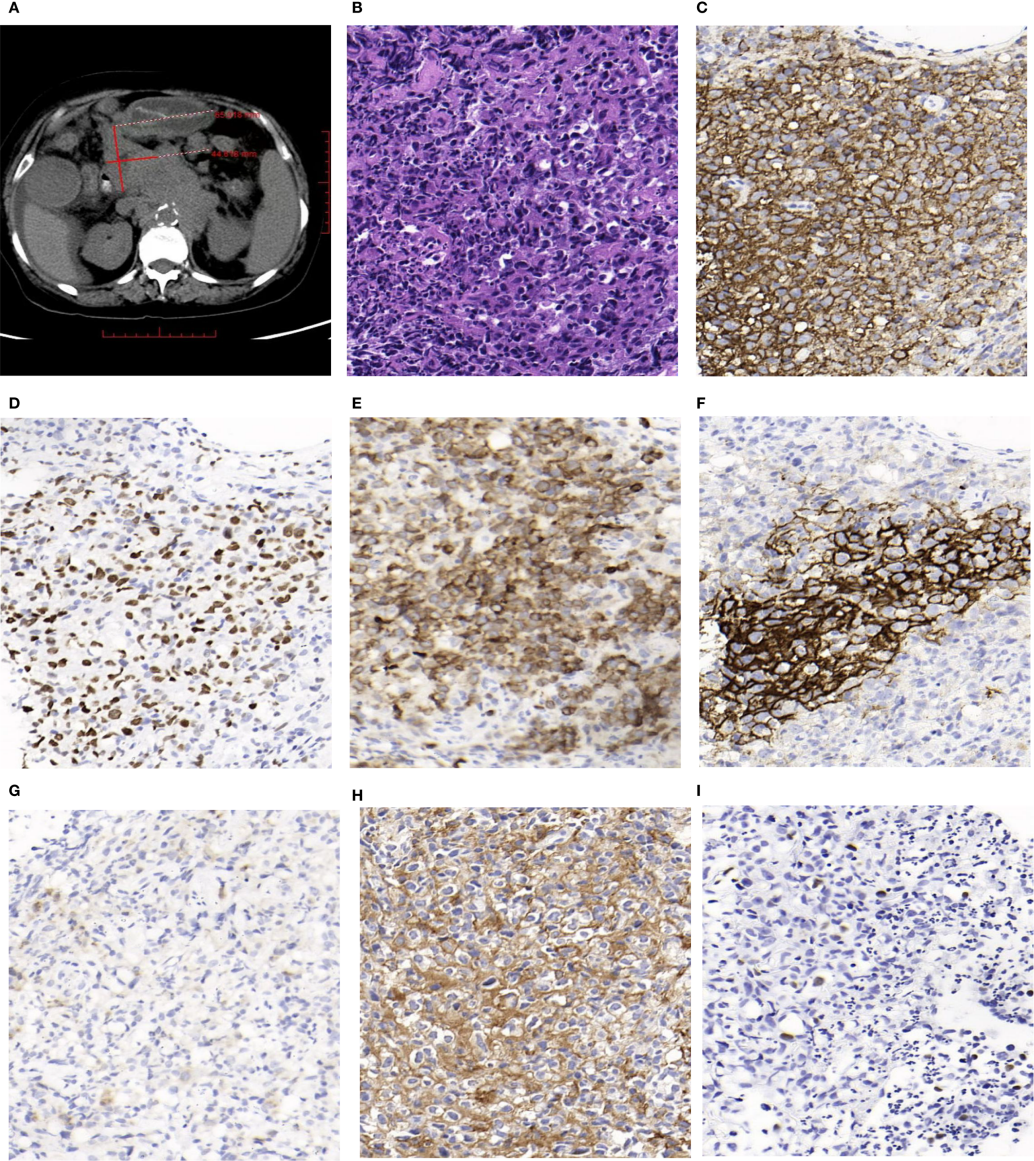
**(A)** Some of enlarged lymph nodes merged into masses,and the largest cross-sectional area measured about 6.5cm×4.5cm(PET-CT). **(B)** Hematoxylin and Eosin (H&E) staining shows a neoplastic follicle composed predominantly of centroblasts, consistent with grade 3B (HE, magnification, ×400). **(C)** Tumor cells in the follicle show diffuse and strong positivity for CD20 (IHC, magnification, ×400). **(D)** Tumor FollicleBCL-6 staining was positive (IHC, magnification, ×400). **(E)** Tumor Follicle BCL-2 staining was positive (IHC, magnification, ×400). **(F)** Tumor FollicleCD21 staining showed irregular FDC meshworks (IHC, magnification, ×400). **(G)** Tumor cells CD30 staining showed individually positive (IHC, magnification, ×400). **(H)** A high proportion of tumor cells (approximately 80%) show strong membranous staining for PD-L1 (IHC, magnification, ×400). **(I)** Some tumor cells showed positive in situ hybridization EBV-EBER (ISH, magnification, ×400).

The patient, a 72-year-old female, was admitted on July 23, 2024, due to abdominal distension and decreased appetite persisting for over six months. CT imaging revealed multiple enlarged lymph nodes of varying sizes in the abdominal cavity, retroperitoneum, and bilateral inguinal regions, as well as several nodules located in the left diaphragmatic peritoneum and the left renal fascia. PET-CT imaging further identified multiple lymph nodes of different sizes near the esophageal hiatus, hepatic hilum, hepatogastric space, retroperitoneum, and abdominal cavity. Some nodes had fused into masses, with the largest measuring approximately 6.5 cm × 4.5 cm in cross-section (**Figure 1A**). Bone marrow flow cytometry showed a significant reduction in the lymphocyte proportion, an inverted CD4/CD8 ratio, and a marked increase in the NK cell population, without notable phenotypic abnormalities. Eosinophils and B progenitor cells were occasionally present. These phenomena could be related to the EBV infection or the patient’s age. The patient subsequently underwent a biopsy of the abdominal mass.

Macroscopy: Two needle biopsy specimens were obtained, each measuring between 1.2 cm and 1.5 cm in length, and approximately 0.1 cm in diameter. Microscopy: Lymphocytes were arranged in nodules with indistinct margins and densely packed structures. These nodules were predominantly composed of centroblasts, which appeared as large cells with abundant eosinophilic or clear cytoplasm and large irregular nuclei with fine chromatin. Mitotic figures were visible, and scattered neutrophils were observed within the nodules (**Figure 1B**). Immunohistochemical Staining: The proliferative lymphocytes within the nodules were positive for CD20 (**Figure 1C**), BCL-6 (**Figure 1D**), and BCL-2 (**Figure 1E**). CD21 staining revealed an irregular follicular dendritic cell (FDC) network (**Figure 1F**). Approximately 1% of the cells expressed CD30 (**Figure 1G**), while PD-L1 was expressed in around 80% of the tumor cells (**Figure 1H**).

PD-L1 expression was assessed via immunohistochemistry using the Ventana platform with a rabbit monoclonal anti-PD-L1 antibody(clone SP263).The evaluation was conducted independently by two pathologists with specialized training. For the sample to be considered evaluable, a minimum of 100 viable tumor cells was required.PD-L1 expression was quantified using the Tumor Proportion Score (TPS), which is determined by the percentage of tumor cells exhibiting any degree of membranous staining relative to all tumor cells in the sample. A specimen was considered PD-L1-positive if at least 1% of tumor cells showed expression.

*In situ* hybridization for EBV-encoded small RNAs (EBER) was positive in some tumor cells (**Figure 1I**).

Pathological Diagnosis: (Abdominal tumor biopsy) Non-Hodgkin lymphoma, WHO classification: Follicular lymphoma, grade IIIB, EBV-positive.

The patient received R-CHOP chemotherapy in the hematology department, combined with anti-CD20 antibody (Rituximab). The treatment yielded a favorable response, achieving complete remission.

## Discussion

FL is a type of non-Hodgkin B-cell lymphoma originating from the germinal center and composed of centrocytes and centroblasts. In Western countries, FL accounts for approximately 22%-35% of all non-Hodgkin lymphomas (NHL), a significantly higher incidence compared to that in Asian countries (6, 7).

EBV-positive follicular lymphoma (EBV+ FL) accounts for approximately 2–6% of all follicular lymphoma cases. Its histological morphology and immunophenotype are similar to those of follicular lymphoma of the same grade (8).Traditionally, the diagnosis of follicular lymphoma has primarily relied on histological morphology (9). Based on the number of centroblasts per high-power field, follicular lymphoma is categorized into grades 1, 2, and 3, with grade 3 further divided into subtypes 3A and 3B. EBV+FL is most frequently classified as grade 3A or 3B.The tumor cells express B-cell-associated antigens (such as CD19, CD20, and CD79a),and are positive for BCL2, BCL6, and MUM1.CD10 is generally positive; however, it may be negative in some cases, particularly in grade 3B follicular lymphoma (10).Furthermore, compared to EBV-negative follicular lymphoma, CD30 expression is more frequently detected in EBV+FL (8).In this case, CD30 positivity was observed in approximately 1% of cells, while PD-L1 positivity reached 80%. Studies (11)have demonstrated that PD-L1 is highly expressed in CD30-positive large cell lymphomas. Moreover, various EBV-encoded proteins and circular RNAs (circRNAs) can regulate PD-L1 expression (12). The upregulation of PD-L1 is regarded as a significant hallmark of EBV-associated lymphoproliferative disorders (13).

Although EBV+FL is generally considered an indolent lymphoma, approximately one-third of cases may undergo histological transformation into a more aggressive form (14). It is well established that EBV infection plays a role in the pathogenesis and progression of several lymphomas, including Burkitt lymphoma, classical Hodgkin lymphoma, post-transplant lymphoproliferative disorders, extranodal NK/T-cell lymphoma, and diffuse large B-cell lymphoma (14, 15). However, the involvement of EBV in the transformation of follicular lymphoma remains a topic of debate. Cases of EBV+FL transforming into diffuse large B-cell lymphoma and classical Hodgkin lymphoma have been reported (15, 16), as well as instances where low-grade follicular lymphoma progressed to high-grade follicular lymphoma following EBV infection (8). Nevertheless, it remains uncertain whether EBV infection represents an early oncogenic event or a later development contributing to disease progression. In addition, high PD-L1 expression has been reported in EBV-positive diffuse large B-cell lymphoma (17), and some cases of follicular lymphoma have also shown high PD-L1 expression (18). However, to date, no cases of high PD-L1 expression specifically in EBV+FL have been reported.

Previous literature (17) has explored the mechanisms behind the high expression of PD-L1 in lymphomas associated with EBV infection. The latent membrane protein(LMP1), encoded by EBV, can activate the transcription factor AP-1, which binds to enhancer elements of the PD-L1 gene, thereby increasing promoter activity and enhancing PD-L1 expression. Additionally, LMP1 can interact with JAK3 and activate the STAT signaling pathway, further promoting PD-L1 promoter activity and expression.LMP1 is a transmembrane protein whose cytoplasmic region contains three key domains—carboxyl-terminal activation regions 1, 2, and 3 (CTR 1–3), which activate nuclear factor κB (NF-κB)signaling pathway, NF-κB can induce PD-L1 expression directly by binding to the PD-L1 promoter or indirectly through post-transcriptional regulation (19, 20).It is worth investigating whether EBV infection in follicular lymphoma also leads to PD-L1 overexpression via LMP1, activating AP-1, JAK/STAT, and NF-κB pathways, thereby enhancing PD-L1 expression. The role of EBV in the tumorigenesis, progression, and potential transformation of follicular lymphoma, as well as its influence on PD-L1 expression, warrants further investigation.

High expression of PD-L1, which is also frequently detected in EBV-associated Post-transplant lymphoproliferative disorders (PTLDs), diffuse large B-cell lymphoma (DLBCL), and Classical Hodgkin lymphoma (CHL).

PTLD is a serious complication occur in immunocompromised hosts after allogeneic hematopoietic cell transplantation (allo-HCT) or solid organ transplantation (SOT).EBV infection precedes PTLD in 90% of patients (21).PTLD is divided into non-destructive PTLD and destructive PTLD. Non-destructive PTLD is an early-stage disease with mild symptoms. The subgroups of destructive PTLD include polymorphic PTLD (lymphocyte proliferation of B and T cells), monomorphic PTLD (DLBCL is the most common), and CHL-like PTLD (22).

In contrast to EBV-negative DLBCL, PD-L1 expression is significantly more common in EBV-positive cases. EBV+ DLBCL upregulates at least three key cellular signaling pathways:AP-1, JAK/STAT, and NF-κB, ultimately leading to PD-L1 overexpression (11, 23). Currently, PD-1/PD-L1 blockade has shown low response rates in trials involving unselected DLBCL patients, but it represents a promising treatment approach for patients with EBV+ DLBCL (11).

In EBV-positive CHL, EBV induces PD-L1 overexpression by activating the transcription factor AP-1 and the JAK/STAT signaling pathway, facilitating immune evasion. Unlike in EBV+ DLBCL, PD-1/PD-L1 inhibitor therapy has proven highly successful in treating CHL (11).

The interaction between PD-1 and PD-L1 inhibits downstream signaling pathways involved in T-cell activation. PD-1/PD-L1 inhibitors disrupt this interaction, weaken the suppression of T-cell activation and stimulate an endogenous anti-tumor immune response (24). PD-1/PD-L1 monoclonal antibodies function by blocking the binding of PD-1 to its ligands (PD-Ls), leading to the reactivation of suppressed T cells *in vivo*. This reactivation enhances the recognition of tumor cells and strengthens the body’s anti-tumor capacity (25).

At present the treatment strategy for EBV+FL generally follows the standard protocols established for follicular lymphoma (26–28):For Stage I disease, radiotherapy is the preferred option for grades 1, 2, and 3A follicular lymphoma. For grade 3B follicular lymphoma, treatment regimens such as R-CHOP, which are used for aggressive lymphomas like DLBCL, are recommended. For Stages II, III, and IV follicular lymphoma, the focus shifts to improving quality of life, alleviating symptoms, and correcting cytopenias. Asymptomatic patients are typically monitored through active surveillance without immediate intervention. Symptomatic advanced-stage follicular lymphoma is managed using anti-CD20 monoclonal antibody (Rituximab) combined with chemotherapy regimens. Autologous hematopoietic stem cell transplantation is the preferred treatment for patients with relapsed disease or those whose lymphoma has transformed into a high-grade form.

Immune checkpoint PD-1/PD-L1 has emerged as an effective immunotherapeutic target for many malignant tumors. We hypothesize that in follicular lymphoma, EBV infection may promote PD-L1 expression through LMP1 overexpression, thereby activating transcription factors AP-1, JAK/STAT, and NF-κB signaling pathways. If this hypothesis holds true, immunotherapy could offer a novel treatment strategy for patients with EBV-positive follicular lymphoma exhibiting high PD-L1 expression. Further studies are needed to validate our hypothesis. The role of EBV in the tumorigenesis, progression, and even transformation of follicular lymphoma, as well as the precise mechanisms by which EBV infection regulates PD-L1 expression, require extensive future investigation.

## Data Availability

The raw data supporting the conclusions of this article will be made available by the authors, without undue reservation.
